# Path-following and semismooth Newton methods for the variational inequality arising from two membranes problem

**DOI:** 10.1186/s13660-019-1955-4

**Published:** 2019-01-05

**Authors:** Shougui Zhang, Yueyue Yan, Ruisheng Ran

**Affiliations:** 1 School of Mathematical Sciences, Chongqing Normal University, Chongqing, P.R. China; 2 College of Computer and Information Science, Chongqing Normal University, Chongqing, P.R. China

**Keywords:** Unilateral contact, Elastic membranes, Variational inequality, Semismooth Newton method, Path-following method

## Abstract

A semismooth Newton method, based on variational inequalities and generalized derivative, is designed and analysed for unilateral contact problem between two membranes. The problem is first formulated as a corresponding regularized problem with a nonlinear function, which can be solved by the semismooth Newton method. We prove the convergence of the method in the function space. To improve the performance of the semismooth Newton method, we use the path-following method to adjust the parameter automatically. Finally, some numerical results are presented to illustrate the performance of the proposed method.

## Introduction

Contact problems appear in many applications in industry and engineering, such as the contact between two elastic membranes [1–7]. This problem describes the equilibrium position of two membranes under the action between them. The membranes cannot interpenetrate and are fixed on the boundary. In this system, there are three unknowns: the position of each membrane and the action of each membrane on the other one [2]. One of the main challenges is the fact that the contact zone is not known in advance and has to be identified. Although the main results on existence and uniqueness can be found in the recent literature [1–4], little attention has been paid to methods for the numerical solution. Therefore, the accurate and efficient numerical simulation of the contact problem is necessary.

We note that different Newton methods have been successfully applied to constrained problems such as complementary problems and variational inequalities in finite or infinite dimensional space [8–17]. Motivated by theoretical and numerical results obtained in recent years, we develop a coupling procedure with combination of semismooth Newton methods (SSNMs) and path-following methods (PFMs) in function space [10, 15, 18]. The essence of the procedure is to reduce the problem to a regularized problem which can be solved by SSNM. The main advantage of SSNM is that the inequality constraints are formulated as a nonlinear system which is equivalent to a sequence of linear systems. However, the convergence speed of SSNM is sensitive to a parameter. To make SSNM more efficient, we propose a path-following strategy to update the parameter automatically for numerical implementation.

The paper is organized as follows: In the next section, we start with the formulation of the contact problem between two elastic membranes and recall some basic results. In Sect. 3, we give a regularized problem and its convergence. The semismooth Newton method is proposed in Sect. 4. A path-following method, based on the semismooth Newton method, is presented in Sect. 5. Finally, in Sect. 6 some numerical results are given to show the performance of our method.

## Problem setting and main results

We consider two elastic membranes in unilateral contact. Throughout the paper, let *Ω* be the bounded and convex domain in $\mathbb{R} ^{2}$ with a Lipschitz boundary *Γ*. For given functions $f_{1}$, $f_{2}$ and nonnegative *g*, the problem is to determine the displacements $u_{1}, u_{2}\in H^{1}(\varOmega )=\{u\in L^{2}(\varOmega ); \partial ^{\alpha }u\in L^{2}(\varOmega ), |\alpha |\le 1\}$. The associated norm is $\|u\|_{H^{1}(\varOmega )}=\{\sum\limits_{|\alpha | \le 1}\|\partial ^{\alpha }u\|^{2}_{L^{2}(\varOmega )}\}^{1/2}$ and the action $\lambda \in L^{2}(\varOmega )=H^{0}(\varOmega )$ (the norm is $\|u\|_{L^{2}(\varOmega )}=\{\int _{\varOmega }|u(x)|^{2}\,dx\}^{1/2}$) such that

2.1 $$\begin{aligned} &-\mu _{1}\Delta u_{1}-\lambda =f_{1}\quad \mbox{in } \varOmega , \end{aligned}$$

2.2 $$\begin{aligned} &-\mu _{2}\Delta u_{2}+\lambda =f_{2}\quad \mbox{in } \varOmega , \end{aligned}$$

2.3 $$\begin{aligned} &u_{1}-u_{2}\geq 0, \qquad \lambda \geq 0,\qquad (u_{1}-u_{2}) \lambda =0\quad \mbox{in }\varOmega , \end{aligned}$$

2.4 $$\begin{aligned} &u_{1}=g\quad \mbox{on }\varGamma , \end{aligned}$$

2.5 $$\begin{aligned} &u_{2}=0\quad \mbox{on }\varGamma , \end{aligned}$$

where the tension coefficients $\mu _{1}>0$ and $\mu _{2}>0$. The solution $(u_{1}, u_{2})$ of (2.1)–(2.5) may be interpreted as a vertical displacement of two membranes stretched by different horizontal heights and pressed together by vertical forces with two densities. In this system, *λ* represents the action of the second membrane on the first one and −*λ* is the reaction. The contact condition (2.3) describes the non-interpenetration of two membranes in *Ω*, and the first membrane can press the second one in the domain that is in contact, i.e. $u_{1}-u_{2}=0$. If there is no contact, i.e. $u_{1}-u_{2}>0$, then the action vanishes with $\lambda =0$. The boundary conditions (2.4) and (2.5) mean that the first membrane is fixed on *Γ* at the height *g* which is a nonnegative function, and the second one is fixed at zero, respectively. (More details are given in [2–4].)

To give the weak formulation of the problem (2.1)–(2.5), we introduce the following space of functions:

$$ H_{g}^{1}(\varOmega ):=\bigl\{ v\in H^{1}(\varOmega ); v=g \mbox{ on }\varGamma \bigr\} , $$

and the convex subsets

$$\begin{aligned}& K_{g}:=\bigl\{ (v_{1},v_{2})\in H^{1}_{g}(\varOmega )\times H^{1}_{0}( \varOmega ); v_{1}-v_{2}\geq 0 \mbox{ a.e. in }\varOmega \bigr\} , \\& H^{\frac{1}{2}}_{+}(\varGamma ):=\bigl\{ v\in H^{\frac{1}{2}}(\varGamma ); v \ge 0 \mbox{ a.e. in }\varGamma \bigr\} , \\& \varLambda :=\bigl\{ v\in L^{2}(\varOmega ); v\ge 0 \mbox{ a.e. in }\varOmega \bigr\} . \end{aligned}$$

For given $(f_{1},f_{2})$ in $L^{2}(\varOmega )\times L^{2}(\varOmega )$ and *g* in $H^{\frac{1}{2}}_{+}(\partial \varOmega )$, we consider the following variational problem: Find $(u_{1},u_{2},\lambda )$ in $H^{1}_{g}(\varOmega )\times H^{1}_{0}(\varOmega )\times \varLambda $, such that

2.6 $$ \textstyle\begin{cases} \sum_{i=1}^{2}\mu _{i}\int _{\varOmega }\nabla u_{i}\cdot \nabla v _{i}\,dx-\int _{\varOmega }\lambda (v_{1}-v_{2})\,dx \\ \quad = \sum_{i=1}^{2}\int _{\varOmega }f_{i}v_{i}\,dx,\quad \forall (v_{1},v _{2})\in H^{1}_{0}(\varOmega )\times H^{1}_{0}(\varOmega ),\\ \int _{\varOmega }(\chi -\lambda )(x)(u_{1}-u_{2})(x)\,dx\geq 0,\quad \forall \chi \in \varLambda , \end{cases} $$

or a variational inequality: Find $(u_{1},u_{2})$ in $K_{g}$, such that

2.7 $$ \sum_{i=1}^{2}\mu _{i} \int _{\varOmega }\nabla u_{i}\cdot \nabla (v _{i}-u_{i})\,dx \ge \sum_{i=1}^{2} \int _{\varOmega }f_{i}(v_{i}-u _{i}) \,dx,\quad \forall (v_{1},v_{2})\in K_{g}. $$

For the above problems, we summarize the main conclusions for the existence and uniqueness as follows (see Proposition 1, Lemma 2 and Proposition 3 in [3]).

### Proposition 2.1

*Problem* (2.6) *is equivalent to problem* (2.1)*–*(2.5), *so that any triple* $(u_{1}^{*},u_{2}^{*},\lambda ^{*})$ *in* $H^{1}_{g}(\varOmega ) \times H^{1}_{0}(\varOmega )\times L^{2}(\varOmega )$ *is a weak solution of* (2.1)*–*(2.5) *if and only if it is a solution of* (2.6).

### Proposition 2.2

*For any solution* $(u_{1}^{*},u_{2}^{*},\lambda ^{*}) \in H^{1}_{g}( \varOmega )\times H^{1}_{0}(\varOmega )\times L^{2}(\varOmega )$ *of problem* (2.6), *the pair* $(u_{1}^{*},u_{2}^{*}) \in H^{1}_{g}(\varOmega )\times H ^{1}_{0}(\varOmega )$ *is a solution of* (2.7).

### Proposition 2.3

*Let data* $(f_{1},f_{2})$ *be in* $L^{2}(\varOmega )\times L^{2}(\varOmega )$ *and* *g* *be in* $H^{\frac{1}{2}}_{+}(\partial \varOmega )$, *then the problem* (2.7) *has a unique solution* $(u_{1}^{*},u_{2}^{*})$ *in* $K_{g}$.

In this paper, we consider the numerical method of the unilateral contact problem.

## Equivalent reformulations

For any $u,v\in L^{2}(\varOmega )$, we define the inner product

$$ \langle u,v\rangle := \int _{\varOmega }u(x)v(x)\,dx, $$

and for any $u,v\in H^{1}(\varOmega )$ the symmetric bilinear form

$$ a(u,v):= \int _{\varOmega }\nabla u(x)\cdot \nabla v(x)\,dx, $$

it follows that the bilinear form $a(\cdot ,\cdot )$ on $H^{1}(\varOmega )\times H^{1}(\varOmega )$ satisfies coercivity and Lipschitz continuity, i.e.

3.1 $$ a(v,v)\geq \alpha \Vert v \Vert _{H_{0}^{1}}^{2} , \qquad a(w,z)\leq \beta \Vert w \Vert _{H ^{1}(\varOmega )} \Vert z \Vert _{H^{1}(\varOmega )}, $$

where $\alpha >0$, $\beta >0$, $v\in H_{0}^{1}(\varOmega )$, $w,z\in H^{1}( \varOmega )$. We also require that the bilinear form $a(\cdot ,\cdot )$ satisfies the weak maximum principle, i.e. for all $v\in H_{0}^{1}( \varOmega )$,

3.2 $$ a\bigl(v,v^{+}\bigr)\leq 0 \quad \mbox{implies}\quad v^{+}=0, $$

where $v^{+}=\max (0,v)$. This property can easily be proved by using the bilinear form $a(\cdot ,\cdot )$.

We note that the condition (2.3) can be rewritten as

3.3 $$ \lambda =\max \bigl(0,\lambda -\gamma (u_{1}-u_{2})\bigr), $$

for any $\gamma >0$ [11, 15]. If we replace (3.3) by

3.4 $$ \lambda =\max \bigl(0,\bar{\lambda }-\gamma (u_{1}-u_{2}) \bigr), $$

where $\bar{\lambda }\in L^{2}(\varOmega )$ is given, then problem (2.1)–(2.5) can be expressed as

3.5 $$ \textstyle\begin{cases} \mu _{1}a(u_{1},v)-\langle \lambda ,v\rangle =\langle f_{1},v\rangle , & \forall v\in H_{0}^{1}(\varOmega ), \\ \mu _{2}a(u_{2},v)+\langle \lambda ,v\rangle =\langle f_{2},v\rangle , & \forall v\in H_{0}^{1}(\varOmega ), \\ \lambda =\max (0,\bar{\lambda }-\gamma (u_{1}-u_{2}))& \mbox{a.e. in } \varOmega . \end{cases} $$

Consequently, the optimization problem for system (3.5) is

3.6 $$ \textstyle\begin{cases} \mbox{Find } (u_{1}, u_{2})\in H^{1}_{g}(\varOmega )\times H^{1}_{0}( \varOmega )\quad \mbox{such that }\\ \min J(\gamma ,u_{1}, u_{2}):=\sum_{i=1}^{2}(\frac{1}{2} \mu _{i} a(u_{i},u_{i})-\langle f_{i},u _{i}\rangle )\\ \hphantom{\min J(\gamma ,u_{1}, u_{2}):=} {} +\frac{1}{2\gamma } \Vert \max (0,\bar{\lambda }-\gamma (u_{1}-u_{2})) \Vert ^{2}. \end{cases} $$

It follows from the uniform convexity of $J(\gamma ,\cdot ,\cdot )$ that the system (3.5) admits a unique solution $(u_{1\gamma },u_{2\gamma }, \lambda _{\gamma })$ for every $\gamma >0$ [11, 15]. To highlight the dependence on *γ*, the solution is denoted by $(u_{1\gamma },u_{2\gamma })$ and the corresponding multiplier by $\lambda _{\gamma }$. In the following theorem, we can show that the problem (2.6) can be approximately formulated as the optimization problem (3.6) with $\gamma \to \infty $.

### Theorem 3.1

*For every* $\bar{\lambda }\in L^{2}(\varOmega )$, *the solutions* $(u_{1\gamma },u_{2\gamma },\lambda _{\gamma })$ *to problem* (3.5) *converge to the solution* $(u_{1}^{*},u_{2}^{*},\lambda ^{*})$ *to problem* (2.6) *in the sense that* $(u_{1\gamma },u_{2\gamma }) \to (u_{1}^{*},u_{2}^{*})$ *strongly in* $H^{1}_{g}(\varOmega )\times H ^{1}_{0}(\varOmega )$ *and* $\lambda _{\gamma }\to \lambda ^{*}$ *weakly in* $H^{-1}(\varOmega )$ *as* $\gamma \to \infty $.

### Proof

From (3.5) we obtain, for any $\gamma >0$,

3.7 $$ \textstyle\begin{cases} \mu _{1} a(u_{1\gamma },u_{1\gamma }-u_{1}^{*})-\langle \lambda _{ \gamma },u_{1\gamma }-u_{1}^{*}\rangle =\langle f_{1},u_{1\gamma }-u _{1}^{*}\rangle , \\ \mu _{2} a(u_{2\gamma },u_{2\gamma }-u_{2}^{*})+\langle \lambda _{ \gamma },u_{2\gamma }-u_{2}^{*}\rangle =\langle f_{2},u_{2\gamma }-u _{2}^{*}\rangle , \end{cases} $$

it follows that

3.8 $$ \begin{aligned}[b] &\mu _{1} a\bigl(u_{1\gamma },u_{1\gamma }-u_{1}^{*} \bigr)+\mu _{2} a\bigl(u_{2\gamma },u_{2\gamma }-u_{2}^{*} \bigr) \\ &\quad = \bigl\langle \lambda _{\gamma },u_{1\gamma }-u_{2\gamma }- \bigl(u_{1}^{*}-u _{2}^{*}\bigr)\bigr\rangle +\bigl\langle f_{1},u_{1\gamma }-u_{1}^{*} \bigr\rangle +\bigl\langle f_{2},u_{2\gamma }-u_{2}^{*} \bigr\rangle . \end{aligned} $$

Note that $\lambda _{\gamma }\geq 0$ from (3.4) and $u_{1}^{*}-u_{2} ^{*}\geq 0$ from (2.3), we have

$$ \begin{aligned} \bigl\langle \lambda _{\gamma },u_{1\gamma }-u_{2\gamma }- \bigl(u_{1}^{*}-u_{2} ^{*}\bigr)\bigr\rangle &= \biggl\langle \lambda _{\gamma },\frac{\overline{\lambda }}{ \gamma }+u_{1\gamma }-u_{2\gamma }- \bigl(u_{1}^{*}-u_{2}^{*}\bigr)- \frac{\overline{ \lambda }}{\gamma }\biggr\rangle \\ &\leq \biggl\langle \lambda _{\gamma },\frac{\overline{\lambda }}{\gamma }+(u _{1\gamma }-u_{2\gamma })-\frac{\overline{\lambda }}{\gamma }\biggr\rangle \\ &= \frac{1}{\gamma }\langle \lambda _{\gamma },\overline{\lambda } \rangle -\frac{1}{\gamma }\bigl\langle \lambda _{\gamma },\overline{\lambda }- \gamma (u_{1\gamma }-u_{2\gamma })\bigr\rangle . \end{aligned} $$

Consequently,

3.9 $$ \bigl\langle \lambda _{\gamma },u_{1\gamma }-u_{2\gamma }- \bigl(u_{1}^{*}-u_{2} ^{*}\bigr)\bigr\rangle \le \frac{1}{\gamma }\langle \lambda _{\gamma },\overline{ \lambda } \rangle -\frac{1}{\gamma } \Vert \lambda _{\gamma } \Vert _{\varOmega } ^{2}, $$

where (3.4) is used. Combining (3.8) and (3.9) we obtain

$$ \begin{aligned} &\mu _{1} a(u_{1\gamma },u_{1\gamma })+ \mu _{2} a(u_{2\gamma },u_{2 \gamma })+\frac{1}{\gamma } \Vert \lambda _{\gamma } \Vert _{\varOmega }^{2} \\ &\quad \leq \mu _{1} a\bigl(u_{1\gamma },u_{1}^{*} \bigr)+\mu _{2} a\bigl(u_{2\gamma },u_{2} ^{*} \bigr)+\bigl\langle f_{1},u_{1\gamma }-u_{1}^{*} \bigr\rangle +\bigl\langle f_{2},u _{2\gamma }-u_{2}^{*} \bigr\rangle +\frac{1}{\gamma }\langle \lambda _{ \gamma },\overline{\lambda }\rangle , \end{aligned} $$

from the coercivity (with positive constants $\alpha _{1}$, $\alpha _{2}$) and the Lipschitz continuity of $a(\cdot ,\cdot )$ it follows that

$$ \alpha _{1}\mu _{1} \Vert u_{1\gamma } \Vert _{H_{g}^{1}}^{2}+\alpha _{2}\mu _{2} \Vert u _{2\gamma } \Vert _{H_{0}^{1}}^{2}+\frac{1}{\gamma } \Vert \lambda _{\gamma } \Vert _{\varOmega }^{2} $$

is uniformly bounded with respect to $\gamma \geq 1$. Clearly $u_{1\gamma }$, $u_{2\gamma }$ are bounded in $H_{g}^{1}$ and $H_{0}^{1}$ respectively, and $\{\lambda _{\gamma }\}_{\gamma \geq 1}$ is bounded in $L^{2}(\varOmega )$ from (3.7) [11]. Then there exist $(\widehat{u}_{1},\widehat{u}_{2},\widehat{\lambda })\in H_{g} ^{1}(\varOmega )\times H_{0}^{1}(\varOmega )\times L^{2}(\varOmega )$ and a sequence $\{u_{1\gamma _{n}},u_{2\gamma _{n}},\lambda _{\gamma _{n}}\}$ with $\lim \gamma _{n}=\infty $ such that

3.10 $$ \lim_{\gamma _{n}\to \infty }(u_{1\gamma _{n}},u_{2\gamma _{n}}, \lambda _{\gamma _{n}})=(\widehat{u}_{1},\widehat{u}_{2}, \widehat{ \lambda }); $$

here we drop subscript *n* with $\gamma _{n}$.

On the other hand, from (3.4) we note that

3.11 $$ \frac{1}{\gamma } \Vert \lambda _{\gamma } \Vert _{\varOmega }^{2}= \gamma \biggl\Vert \mathrm{max}\biggl(0,\frac{\overline{\lambda }}{\gamma }-(u_{1\gamma }-u _{2\gamma })\biggr) \biggr\Vert _{\varOmega }^{2}. $$

Using the above equality and $\lim_{\gamma \to \infty }\frac{1}{ \gamma }\|\lambda _{\gamma }\|^{2}_{L^{2}(\varOmega )}=0$, we have $\lim_{\gamma \to \infty }(u_{1\gamma }-u_{2\gamma })= \widehat{u}_{1}-\widehat{u}_{2}\ge 0$ a.e. on *Ω*. Since $(u_{1}^{*},u_{2}^{*},\lambda ^{*})$ is the unique solution of the problem (2.6), from (3.7) we also have

3.12 $$ \textstyle\begin{cases} \mu _{1} a(u_{1\gamma }-u_{1}^{*},u_{1\gamma }-u_{1}^{*})-\langle \lambda _{\gamma }-\lambda ^{*},u_{1\gamma }-u_{1}^{*}\rangle =0, \\ \mu _{2} a(u_{2\gamma }-u_{2}^{*},u_{2\gamma }-u_{2}^{*})+\langle \lambda _{\gamma }-\lambda ^{*},u_{2\gamma }-u_{2}^{*}\rangle =0, \end{cases} $$

then

3.13 $$ \mu _{1} a\bigl(u_{1\gamma }-u_{1}^{*},u_{1\gamma }-u_{1}^{*} \bigr)+\mu _{2} a\bigl(u _{2\gamma }-u_{2}^{*},u_{2\gamma }-u_{2}^{*} \bigr)=\bigl\langle \lambda _{\gamma }-\lambda ^{*},u_{1\gamma }-u_{2\gamma }- \bigl(u_{1}^{*}-u_{2}^{*}\bigr)\bigr\rangle . $$

Using (3.9) and Young’s inequality, we have

3.14 $$ \bigl\langle \lambda _{\gamma },u_{1\gamma }-u_{2\gamma }- \bigl(u_{1}^{*}-u_{2} ^{*}\bigr)\bigr\rangle \leq \frac{1}{2\gamma } \Vert \overline{\lambda } \Vert _{\varOmega } ^{2}. $$

Hence

$$ \begin{aligned} 0 &\leq \alpha _{1}\mu _{1} \bigl\Vert u_{1\gamma }-u_{1}^{*} \bigr\Vert _{H_{0}^{1}(\varOmega )}^{2}+\alpha _{2}\mu _{2} \bigl\Vert u_{2\gamma }-u_{2}^{*} \bigr\Vert _{H_{0}^{1}(\varOmega )}^{2} \\ &\leq \mu _{1} a_{1}\bigl(u_{1\gamma }-u_{1}^{*},u_{1\gamma }-u_{1}^{*} \bigr)+ \mu _{2} a_{2}\bigl(u_{2\gamma }-u_{2}^{*},u_{2\gamma }-u_{2}^{*} \bigr) \\ &=\bigl\langle \lambda _{\gamma }-\lambda ^{*},u_{1\gamma }-u_{2\gamma }- \bigl(u _{1}^{*}-u_{2}^{*}\bigr)\bigr\rangle \\ &=\bigl\langle \lambda _{\gamma },u_{1\gamma }-u_{2\gamma }- \bigl(u_{1}^{*}-u _{2}^{*}\bigr)\bigr\rangle -\bigl\langle \lambda ^{*},u_{1\gamma }-u_{2\gamma }- \bigl(u _{1}^{*}-u_{2}^{*}\bigr)\bigr\rangle \\ &\leq \frac{1}{2\gamma } \Vert \overline{\lambda } \Vert _{\varOmega }^{2}-\bigl\langle \lambda ^{*},u_{1\gamma }-u_{2\gamma }- \bigl(u_{1}^{*}-u_{2}^{*}\bigr)\bigr\rangle . \end{aligned} $$

Note that $\lambda ^{*}\ge 0$, $\widehat{u}_{1}-\widehat{u}_{2}\ge 0$ and $u_{1}^{*}-u_{2}^{*}\ge 0$, we thus have

$$\begin{aligned} 0 &\leq \lim_{\gamma \to \infty } \sup \bigl(\alpha _{1}\mu _{1} \bigl\Vert u _{1\gamma }-u_{1}^{*} \bigr\Vert _{H_{0}^{1}(\varOmega )}^{2}+\alpha _{2}\mu _{2} \bigl\Vert u _{2\gamma }-u_{2}^{*} \bigr\Vert _{H_{0}^{1}(\varOmega )}^{2}\bigr) \\ &\leq \lim_{\gamma \to \infty }\biggl(\frac{1}{2\gamma } \Vert \overline{ \lambda } \Vert _{\varOmega }^{2}+\bigl\langle \lambda ^{*},u_{1}^{*}-u_{2}^{*} \bigr\rangle -\bigl\langle \lambda ^{*},u_{1\gamma }-u_{2\gamma } \bigr\rangle \biggr) \\ &=-\lim_{\gamma \to \infty }\bigl\langle \lambda ^{*},\widehat{u} _{1}-\widehat{u}_{2}\bigr\rangle \\ &\leq 0. \end{aligned}$$

This implies that

$$ \lim_{\gamma \to \infty } u_{1\gamma }=u_{1}^{*},\lim _{\gamma \to \infty } u_{2\gamma }=u_{2}^{*}. $$

So we obtain from (3.10)

$$ \widehat{u}_{1}=u_{1}^{*},\widehat{u}_{2}=u_{2}^{*}. $$

Taking the limit $\gamma \to \infty $ in

$$ \textstyle\begin{cases} \mu _{1}a(u_{1\gamma },v)-\langle \lambda _{\gamma },v\rangle =\langle f_{1},v\rangle , \quad \forall v\in H_{g}^{1}(\varOmega ), \\ \mu _{2} a(u_{2\gamma },v)+\langle \lambda _{\gamma },v\rangle =\langle f_{2},v\rangle , \quad \forall v\in H_{0}^{1}(\varOmega ), \end{cases} $$

yields

3.15 $$ \textstyle\begin{cases} \mu _{1}a(u_{1}^{*},v)-\langle \widehat{\lambda },v\rangle =\langle f _{1},v\rangle , \quad \forall v\in H_{g}^{1}(\varOmega ), \\ \mu _{2} a(u_{2}^{*},v)+\langle \widehat{\lambda },v\rangle =\langle f _{2},v\rangle , \quad \forall v\in H_{0}^{1}(\varOmega ). \end{cases} $$

Comparing (3.15) and (3.5) shows that $\lambda ^{*}$ and *λ̂* satisfy the same equation. Consequently, we have $\lambda ^{*}=\widehat{\lambda }$ in $H^{-1}(\varOmega )$. It follows from the uniqueness of the solution variables $(u_{1}^{*},u_{2}^{*},\lambda ^{*})$ that the whole family $\{(u_{1\gamma },u_{2\gamma }, \lambda _{\gamma })\}$ converges in the sense stated in the theorem. □

## Semismooth Newton method

This section is devoted to the discussion of an iterative algorithm for solving (3.5). Note that the direct application of a Newton algorithm is impeded by the fact that the max-function is not differentiable. Alternatively we shall apply a semismooth Newton method to the mapping $F: L^{2}(\varOmega ) \to L^{2}( \varOmega )$ defined by

$$ F(\lambda )=\lambda -\max \bigl(0,\bar{\lambda }-\gamma \bigl(u_{1}( \lambda )-u _{2}(\lambda )\bigr)\bigr). $$

We now briefly recall those facts on semismooth Newton methods which are relevant for the present context [11, 13, 15].

### Definition 4.1

The mapping $F:D \subset X \to Z $ is called generalized-differentiable on the open subset $U\subset D$ if there exists a family of generalized derivatives $G: U\rightarrow L(X,Z)$ such that

$$ \lim_{h \to \infty } \frac{1}{ \Vert h \Vert } \bigl\Vert F(x+h)-F(x)-G(x+h)h \bigr\Vert =0, $$

for every $x\in U$.

### Theorem 4.1

*Suppose that* $x^{*}\in D$ *is a solution to* $F(x)=0$ *and that* *F* *is Newton*-*differentiable in an open neighborhood* *U* *containing* $x^{*}$ *and that* ${\|G(x)^{-1}\|:x\in U}$ *is bounded*. *Then the Newton*-*iteration* $x_{k+1}=x_{k}-G(x_{k})^{-1}F(x_{k})$ *converges superlinearly to* $x^{*}$ *provided that* $\|x_{0}-x^{*}\|$ *is sufficiently small*.

Let us consider Newton-differentiability of the max-operation. We introduce candidates for the generalized derivatives of the form

$$ G_{m}(y) (x)= \textstyle\begin{cases} 1 & y(x)>0, \\ 0 & y(x)\le 0, \end{cases} $$

where $y\in X$.

### Proposition 4.1

*The mapping* $\max (0,\cdot )$ *with* $1\leq p< q<\infty $ *is Newton*-*differentiable on* $L^{q}(\varOmega )$ *and* $G_{m}$ *is a generalized derivative*.

Now we can describe our semismooth Newton method for the problem (3.5) as follows.

### Algorithm 1

(SSNM)

1. Choose initial triple $(u_{1}^{(0)},u_{2}^{(0)},\bar{\lambda }) \in H_{g}^{1}(\varOmega ) \times H_{0}^{1}(\varOmega )\times L^{2}(\varOmega )$ and big enough $\gamma >0$, set $k=0$.
2. Set $\mathcal{A}_{k+1}=\{x\in \varOmega :\overline{\lambda }-\gamma (u_{1}^{(k)}-u_{2}^{(k)})>0\}$, $\mathcal{I}_{k+1}=\varOmega \setminus \mathcal{A}_{k+1}$.
3. Determine $(u_{1}^{(k+1)},u_{2}^{(k+1)})\in H_{g}^{1}(\varOmega ) \times H_{0}^{1}(\varOmega )$ such that
  4.1 $$ \textstyle\begin{cases} \mu _{1} a(u_{1}^{(k+1)},v)-\langle \lambda ^{(k+1)},v\rangle =(f_{1},v), \quad \forall v\in H_{0}^{1}(\varOmega ), \\ \mu _{2} a(u_{2}^{(k+1)},v)+\langle \lambda ^{(k+1)},v\rangle =(f_{2},v), \quad \forall v\in H_{0}^{1}(\varOmega ). \end{cases} $$
4. Set
  $$ \lambda ^{(k+1)}= \textstyle\begin{cases} 0 &\mbox{on } \mathcal{I}_{k+1}, \\ \overline{\lambda }-\gamma (u_{1}^{(k+1)}-u_{2}^{(k+1)}) &\mbox{on } \mathcal{A}_{k+1}. \end{cases} $$
5. Stop or set $k:=k+1$ and go to (2).

Following the analysis in [11, 15], we have the same results.

### Proposition 4.2

*If* $\mathcal{A}_{k+1}=\mathcal{A}_{k}$ ($k\geq 1$), *then* $(u_{1}^{(k)},u _{2}^{(k)},\lambda ^{(k)})$ *is the solution to* (4.1).

### Proof

Consider $\mathcal{A}_{k+1}=\mathcal{A}_{k}$, from (4.1) we have

$$ \textstyle\begin{cases} \mu _{1} a(u_{1}^{(k+1)},v)-\langle \overline{\lambda }-\gamma (u_{1} ^{(k+1)}-u_{2}^{(k+1)}),\chi _{\mathcal{A}_{k}}v\rangle =(f_{1},v),\quad \forall v\in H_{0}^{1}(\varOmega ), \\ \mu _{1} a(u_{1}^{(k)},v)-\langle \overline{\lambda }-\gamma (u_{1} ^{(k)}-u_{2}^{(k)}),\chi _{\mathcal{A}_{k}}v\rangle =(f_{1},v),\quad \forall v\in H_{0}^{1}(\varOmega ). \end{cases} $$

Subtracting the second equation from the first one we can get

4.2 $$ \mu _{1}\mu _{2} a\bigl(u_{1}^{(k+1)}-u_{1}^{(k)},v \bigr)=-\gamma \mu _{2}\bigl\langle u_{1}^{(k+1)}-u_{2}^{(k+1)}- \bigl(u_{1}^{(k)}-u_{2}^{(k)}\bigr), \chi _{\mathcal{A}_{k}}v\bigr\rangle . $$

Similarly, we also have

4.3 $$ \mu _{1}\mu _{2} a\bigl(u_{2}^{(k+1)}-u_{2}^{(k)},v \bigr)=\gamma \mu _{1}\bigl\langle u _{1}^{(k+1)}-u_{2}^{(k+1)}- \bigl(u_{1}^{(k)}-u_{2}^{(k)}\bigr), \chi _{\mathcal{A}_{k}}v\bigr\rangle . $$

Subtracting (4.3) from (4.2), it follows that

4.4 $$ \begin{aligned}[b] &\mu _{1}\mu _{2} a \bigl(u_{1}^{(k+1)}-u_{2}^{(k+1)}- \bigl(u_{1}^{(k)}-u_{2}^{(k)}\bigr),v\bigr) \\ &\quad =-\gamma (\mu _{1}+\mu _{2})\bigl\langle u_{1}^{(k+1)}-u_{2}^{(k+1)}-\bigl(u _{1}^{(k)}-u_{2}^{(k)}\bigr),\chi _{\mathcal{A}_{k}}v\bigr\rangle . \end{aligned} $$

Setting $v=u_{1}^{(k+1)}-u_{2}^{(k+1)}-(u_{1}^{(k)}-u_{2}^{(k)})$ and using the coercivity of $a(\cdot ,\cdot )$, we then have

$$ \mu _{1}\mu _{2}\alpha \bigl\Vert u_{1}^{(k+1)}-u_{2}^{(k+1)}- \bigl(u_{1}^{(k)}-u_{2} ^{(k)}\bigr) \bigr\Vert \leq 0, $$

which implies that $u_{1}^{(k+1)}-u_{2}^{(k+1)}=u_{1}^{(k)}-u_{2}^{(k)}$. And we derive from (3.4) that $\lambda ^{(k+1)}=\lambda ^{(k)}$. Using the ellipticity of bilinear form $a(u,v)$ and $\mathcal{A}_{k+1}=\mathcal{A}_{k}$ we see that (4.1) has unique solution. It means that $u_{1}^{(k+1)}=u_{1} ^{(k)}$, $u_{2}^{(k+1)}=u_{2}^{(k)}$. From what has been discussed above, it follows that $(u_{1}^{(k)},u_{2}^{(k)},\lambda ^{(k)})$ is the unique solution to (4.1). □

### Proposition 4.3

*For the sequence* $\{(u_{1}^{(k)},u_{2}^{(k)})\}$ *generated by Algorithm* 1 (*SSNM*), *it follows that* $u_{1}^{(k)}-u_{2}^{(k)}\leq u_{1}^{(k+1)}-u _{2}^{(k+1)}$ ($k\geq 1$) *a*.*e*. *on* *Ω*.

### Proof

We denote $\delta u=\delta u_{2}-\delta u_{1}$, where $\delta u_{1}=u_{1}^{(k+1)}-u_{1}^{(k)}$, $\delta u_{2}=u_{2}^{(k+1)}-u _{2}^{(k)}$ for $k\geq 1$. From (3.5) we have

$$\begin{aligned}& \mu _{1} a\bigl(\delta u_{1},\delta u^{+}\bigr)- \bigl\langle \lambda _{k+1}-\lambda _{k},\delta u^{+}\bigr\rangle =0, \\& \mu _{2} a\bigl(\delta u_{2},\delta u^{+}\bigr)+ \bigl\langle \lambda _{k+1}-\lambda _{k},\delta u^{+}\bigr\rangle =0. \end{aligned}$$

This yields

$$ \mu _{1}\mu _{2} a\bigl(\delta u,\delta u^{+} \bigr)=-(\mu _{1}+\mu _{2})\bigl\langle \lambda _{k+1}-\lambda _{k},\delta u^{+}\bigr\rangle . $$

From Algorithm 1 we have

$$ \lambda _{k+1}(x)-\lambda _{k}(x) \textstyle\begin{cases} =0 & \mbox{for }x\in \mathcal{I}_{k+1}\cap \mathcal{I}_{k}, \\ =\gamma \delta u(x) & \mbox{for }x\in \mathcal{A}_{k+1}\cap \mathcal{A}_{k}, \\ =(-\overline{\lambda }+\gamma (u_{1}^{(k)}-u_{2}^{(k)}))(x)\geq 0 & \mbox{for }x\in \mathcal{I}_{k+1}\cap \mathcal{A}_{k}, \\ >\gamma \delta u(x) & \mbox{for }x\in \mathcal{A}_{k+1}\cap \mathcal{I}_{k}. \end{cases} $$

It follows that $(\lambda _{k+1}-\lambda _{k},\delta u^{+})\geq 0$, we obtain

$$ a\bigl(\delta u,\delta u^{+}\bigr)=-(\mu _{1}+\mu _{2})\bigl\langle \lambda _{k+1}- \lambda _{k}, \delta u^{+}\bigr\rangle \leq 0. $$

Consequently $\delta u^{+}=0$, and the result follows from (3.2). □

### Proposition 4.4

*For all* $\mathcal{I}_{k} (k \ge 1)$ *generated by Algorithm* 1 (*SSNM*), *it follows that* $\mathcal{I}_{k}\subset \mathcal{I}_{k+1}$.

### Proof

Suppose that $\mathcal{I}_{k+1}\subseteq \mathcal{I}_{k}$, then there exists a non-empty set $S\subset \varOmega $ and $S=\mathcal{A} _{k+1}\cap \mathcal{I}_{k}$. From $x\in \mathcal{I}_{k}$ it follows that $(\overline{\lambda }-\gamma (u_{1}^{(k-1)}-u_{2}^{(k-1)}))(x) \le 0$ and by Proposition 4.3 $(\overline{\lambda }-\gamma (u_{1}^{(k)}-u _{2}^{(k)}))(x)\le 0$. On the other hand $x\in \mathcal{A}_{k+1}$, and hence $(\overline{\lambda }-\gamma (u_{1}^{(k)}-u_{2}^{(k)}))(x)> 0$. This gives the desired contradiction. □

### Proposition 4.5

*For every* $k \ge 1$ *we have* $0\le \lambda ^{(k+1)}\le \lambda ^{(k)}$.

### Proof

From Proposition 4.3 we have

$$ u_{1}^{(k)}-u_{2}^{(k)}\leq u_{1}^{(k+1)}-u_{2}^{(k+1)}. $$

Moreover, $\lambda ^{(k+1)}$ in Algorithm 1 is defined by

$$ \lambda ^{(k+1)}= \textstyle\begin{cases} 0 &\mbox{on } \mathcal{I}_{k+1}, \\ \overline{\lambda }-\gamma (u_{1}^{(k+1)}-u_{2}^{(k+1)}) &\mbox{on } \mathcal{A}_{k+1}. \end{cases} $$

This means that the sequence $\{\lambda _{k}\}$ is monotonically decreasing and bounded. □

### Theorem 4.2

*For every* $\gamma >0$ *we have*

$$ \lim_{k \to \infty }\bigl(u_{1}^{(k)},u_{2}^{(k)}, \lambda _{k}\bigr)=(u_{1\gamma },u_{2\gamma },\lambda _{\gamma }) $$

*in* $H_{g}^{1}(\varOmega )\times H_{0}^{1}(\varOmega )\times L^{2}(\varOmega )$.

### Proof

Let $u^{(k)}=u_{2}^{(k)}-u_{1}^{(k)}$, then it follows from Proposition 4.3 and Proposition 4.5 that the sequences $\{u_{k}\}_{k=1} ^{\infty }$ and $\{\lambda _{k}\}_{k=1}^{\infty }$ are decreasing pointwise almost everywhere and are uniformly bounded in $H^{1}( \varOmega )$ and $L^{2}(\varOmega )$, respectively. Hence there exist $\widehat{u}\in H^{1}(\varOmega )$ and $\widehat{\lambda }\in L^{2}( \varOmega )$ such that $\lim_{k\to \infty } u^{(k)}=\widehat{u}$ a.e. and $\lim_{k\to \infty }\lambda ^{(k)}=\widehat{\lambda }$ a.e. Note that $\mathcal{I}_{k}\subset \mathcal{I}_{k+1}$ from Proposition 4.4 and $\lambda ^{(k)}=0$ on $\mathcal{I}_{k}$, we have $\widehat{\lambda }=0$ on $\mathcal{I}=\bigcup_{k=1}^{\infty } \mathcal{I}_{k}$. In this case, we have $(\overline{\lambda }-\gamma \widehat{u})(x)\leq 0$. On the other hand, $\widehat{\lambda }=\overline{ \lambda }-\gamma \widehat{u}$ on $\mathcal{A}=\bigcap_{k=1} ^{\infty }\mathcal{A}_{k}$ where such that $(\overline{\lambda }- \gamma u^{(k)})(x)>0$ for all *k* and hence $(\overline{\lambda }- \gamma \widehat{u})(x)\geq 0$. Consequently, we have $\widehat{\lambda }=\mathrm{max}(0,\overline{\lambda }-\gamma \widehat{u})$. Using Lebesgue’s bounded convergence theorem, it follows that $\lim_{k\to \infty }\lambda ^{(k)}=\widehat{\lambda }$ in $L^{2}(\varOmega )$. Take the limit in the system

$$ \textstyle\begin{cases} \mu _{1} a(u_{1}^{(k)},v)-(\lambda ^{(k)},v)=(f_{1},v), \quad \forall v \in H_{g}^{1}(\varOmega ), \\ \mu _{2} a(u_{2}^{(k)},v)+(\lambda ^{(k)},v)=(f_{2},v),\quad \forall v\in H _{0}^{1}(\varOmega ), \end{cases} $$

we obtain

$$ \textstyle\begin{cases} \mu _{1} a(\widehat{u}_{1},v)-(\widehat{\lambda },v)=(f_{1},v), \quad \forall v\in H_{g}^{1}(\varOmega ), \\ \mu _{2} a(\widehat{u}_{2},v)+(\widehat{\lambda },v)=(f_{2},v), \quad \forall v\in H_{0}^{1}(\varOmega ), \\ \widehat{\lambda }=\mathrm{max}(0,\overline{\lambda }-\gamma \widehat{u}), \end{cases} $$

where $\lim_{k\to \infty }u_{1}^{(k)}=\widehat{u}_{1}$, $\lim_{k\to \infty }u_{2}^{(k)}=\widehat{u}_{2}$. Considering that the solution of the system (3.5) is unique, we have $(\widehat{u}_{1}, \widehat{u}_{2},\widehat{\lambda })=(u_{1\gamma },u_{2\gamma }, \lambda _{\gamma })$; that is $\lim_{k\to \infty }(u_{1}^{(k)},u _{2}^{(k)},\lambda ^{(k)})=(u_{1\gamma },u_{2\gamma },\lambda _{\gamma })$. Then the result follows from the coercivity of $a(\cdot ,\cdot )$. □

## Path-following method

As in Theorem 3.1, the solution converge only if $\gamma \to \infty $. If the parameter *γ* is too small, the SSNM converges slowly. On the contrary, if the *γ* is too big, it may result in a badly conditioned problem. Therefore, the SSNM needs a continuous procedure with respect to *γ*. We mention that path-following schemes for problems posed in function space have become popular in recent years. Such a procedure has already been applied to obstacles and contact problems in linear elasticity [10, 15, 18].

In this section, we give a brief review of path-following method for treating semismooth Newton methods, which can be applied to the unilateral contact problem between membranes. We introduce the primal infeasibility measure $\rho _{F}$, and the complementarity measure $\rho _{C}$ for the $(k + 1)$th iterate as follows:

$$\begin{aligned}& \rho _{F}^{(k+1)}:= \int _{\varOmega }\bigl(u_{1}^{(k+1)}-u_{2}^{(k+1)} \bigr)^{-}\,dx, \\& \rho _{C}^{(k+1)}:= \int _{\mathcal{I}_{k+1}}\bigl(u_{1}^{(k+1)}-u_{2}^{(k+1)} \bigr)^{-}\,dx+ \int _{\mathcal{A}_{k+1}}\bigl(u_{1}^{(k+1)}-u_{2}^{(k+1)} \bigr)^{+}\,dx. \end{aligned}$$

Then we can update the parameter *γ* by

5.1 $$ \gamma ^{(k+1)}=\max \biggl(\gamma ^{(k)}\max \biggl(\tau , \frac{\rho _{F}^{(k+1)}}{ \rho _{C}^{(k+1)}}\biggr), \frac{1}{(\max (\rho _{F}^{(k+1)},\rho _{C}^{(k+1)}))^{q}}\biggr), $$

where $\tau >1$ and $q\ge 1$. So we obtain the following path-following method.

### Algorithm 2

(PFM)

1. Choose $(u_{1}^{(0)},u_{2}^{(0)})\in H_{g}^{1}(\varOmega ) \times H _{0}^{1}(\varOmega )$, $\bar{\lambda }\in L^{2}(\varOmega )$ and $\gamma ^{(0)}>0$, set $k=0$.
2. Set $\mathcal{A}_{k+1}=\{x\in \varOmega :\overline{\lambda }-\gamma ^{(k)} (u_{1}^{(k)}-u_{2}^{(k)})>0\}$, $\mathcal{I}_{k+1}=\varOmega \setminus \mathcal{A}_{k+1}$.
3. Determine $(u_{1}^{(k+1)},u_{2}^{(k+1)})\in H_{g}^{1}(\varOmega ) \times H_{0}^{1}(\varOmega )$ such that
  5.2 $$ \textstyle\begin{cases} \mu _{1} a(u_{1}^{(k+1)},v)-\langle \lambda ^{(k+1)},v\rangle =(f_{1},v), \quad \forall v\in H_{0}^{1}(\varOmega ), \\ \mu _{2} a(u_{2}^{(k+1)},v)+\langle \lambda ^{(k+1)},v\rangle =(f_{2},v), \quad \forall v\in H_{0}^{1}(\varOmega ). \end{cases} $$
4. Set
  $$ \lambda ^{(k+1)}= \textstyle\begin{cases} 0 &\mbox{on }\mathcal{I}_{k+1}, \\ \overline{\lambda }-\gamma ^{(k)} (u_{1}^{(k+1)}-u_{2}^{(k+1)}) & \mbox{on } \mathcal{A}_{k+1}. \end{cases} $$
5. Stop or update $\gamma ^{(k)}$ according to (5.1), set $k:=k+1$ and go to (2).

In our numerical test, we take $\tau =2$ and $q=2$ in (5.1).

## Numerical results

To demonstrate the efficiency and accuracy of the proposed method, we present some numerical results in this section. In this example, we consider the problem in the domain $\varOmega =(-1,1)\times (-1,1)$ with $\mu _{1}=\mu _{2}=1$ and

$$\begin{aligned}& f_{1}(r,\theta )= \textstyle\begin{cases} -10h & r\leq \frac{1}{\sqrt{2}}, \\ -8h & r\ge \frac{1}{\sqrt{2}}, \end{cases}\displaystyle \\& f_{2}(r,\theta )= \textstyle\begin{cases} -6h & r\leq \frac{1}{\sqrt{2}}, \\ -h\frac{1+8g-18r^{2}}{r}\frac{\sqrt{2}}{\sqrt{2}-1} & r\ge \frac{1}{ \sqrt{2}}, \end{cases}\displaystyle \end{aligned}$$

where $0\leq \theta \leq 2\pi $, $h=0.05$ and $r=\sqrt{x^{2}+y^{2}}$ ($x=r \cos \theta $, $y=r\sin \theta $). For this problem, the exact solution in the domain *Ω* is given by

$$\begin{aligned}& u_{1}(r,\theta )=h\bigl(2r^{2}-1\bigr), \\& u_{2}(r,\theta )= \textstyle\begin{cases} h(2r^{2}-1) &r\leq \frac{1}{\sqrt{2}}, \\ h(1-r)(2r^{2}-1)\frac{\sqrt{2}}{\sqrt{2}-1} &r\ge \frac{1}{ \sqrt{2}}, \end{cases}\displaystyle \\& \lambda (r,\theta )= \textstyle\begin{cases} 2h & r\leq \frac{1}{\sqrt{2}}, \\ 0 & r\ge \frac{1}{\sqrt{2}}. \end{cases}\displaystyle \end{aligned}$$

From the analytic solution, we can easily obtain the boundary condition on *Γ* [4].

**Figure 1:**
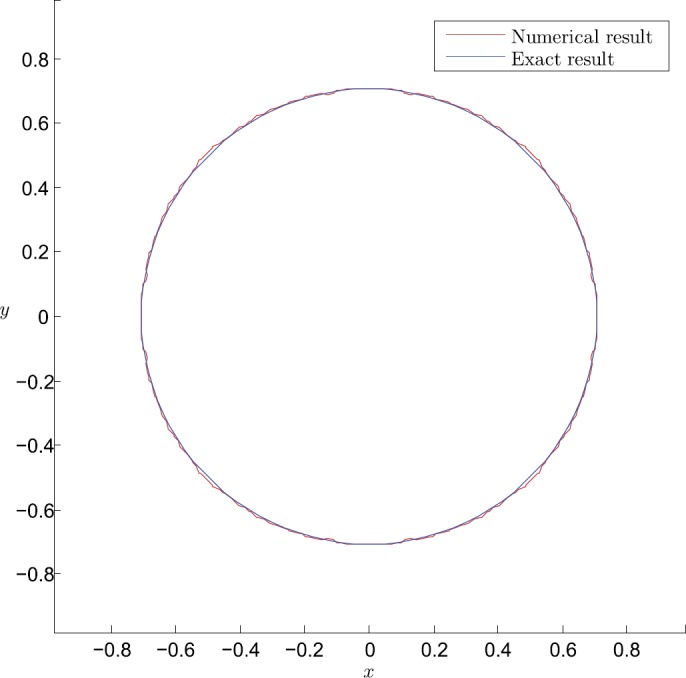
The comparison between numerical result and exact result

To simplify the numerical process, we use linear finite elements to discretize problem (5.2) and solve the corresponding linear system in Matlab codes. We first apply our method to this problem with the number of element $N=800\times 800$ and $\rho =10\mbox{,}000$. Figure 1 plots the numerical and the exact results for the boundary of the contact zone $u_{1}=u_{2}$. It can be seen that our results are in good agreement with the exact contact zone.

**Figure 2:**
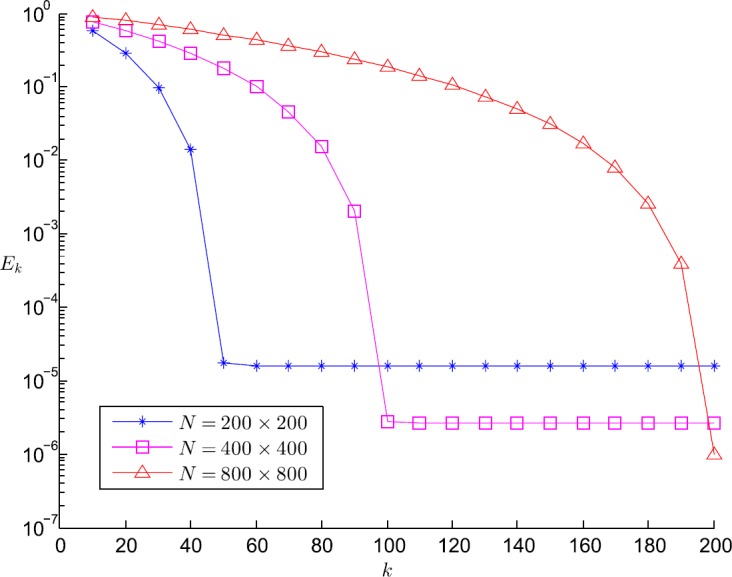
Evolution of $E_{k}$ with respect to *k* for different *N*

Next, we investigate the convergence behavior of our method. In Fig. 2 we provide the evolution of the relative error,

$$ E_{k}:=\frac{ \Vert u_{1}-u_{1h}^{(k)} \Vert ^{2}_{L^{2}(\varOmega )}+ \Vert u_{2}-u _{2h}^{(k)} \Vert ^{2}_{L^{2}(\varOmega )}}{ \Vert u_{1} \Vert ^{2}_{L^{2}(\varOmega )}+ \Vert u _{2} \Vert ^{2}_{L^{2}(\varOmega )}}, $$

with respect to the iteration index *k* for $N=200\times 200$, $N=400\times 400$ and $N=800\times 800$. We note that our method converges for different mesh sizes. Although the number of iterations increases for increasing number of elements, the finer grid yields the smaller relative error.
